# Geospatial Distribution of Food Environments and Their Association with Sociodemographic Factors in Two Mid-Sized Chilean Cities

**DOI:** 10.3390/nu18132131

**Published:** 2026-07-01

**Authors:** Anna C. Pinheiro, Salvador Ayala, Daiana Quintiliano Scarpelli-Dourado, María Rita Marques de Oliveira, Jacqueline Araneda-Flores

**Affiliations:** 1 Carrera de Nutrición y Dietética, Facultad de Medicina-Clínica Alemana, Universidad del Desarrollo, Avenida La Plaza, 680, Las Condes, Santiago 7610658, Chile; apinheiro@udd.cl (A.C.P.); d.quintiliano@udd.cl (D.Q.S.-D.); 2 Centro de Epidemiología y Políticas de Salud, Facultad de Medicina-Clínica Alemana, Universidad del Desarrollo, Avenida La Plaza, 680, Las Condes, Santiago 7610658, Chile; salvadorayala@udd.cl; 3 Faculty of Medical Sciences, Institute of Biosciences, São Paulo State University (UNESP), Rua Prof. Dr. Antônio Celso Wagner Zanin, Botucatu 18618-689, SP, Brazil; 4 Department of Nutrition and Public Health, Faculty of Health and Food Sciences, University of Bío-Bío, Campus Fernando May, Av. Andrés Bello 720, Chillán 3810189, Chile

**Keywords:** food environment, food availability, geographic information systems, Chile

## Abstract

Background/Objectives: The spatial interaction between obesity, diet-related chronic diseases, premature mortality, food environments, and social determinants of health is a global concern. This study aimed to analyze the spatial distribution of healthy and unhealthy food outlets in two medium-sized cities in southern Chile. Methods: A cross-sectional observational study was conducted to characterize the food environment in two medium-sized cities in Chile. Food outlets were geolocated and classified as healthy, regular, or unhealthy, and into four food environment categories (institutional outlets, informal markets, formal markets, and restauration) using validated instruments. Results: Of 1972 identified sites, 1949 were geolocated. Among these, 246 (12.6%) were classified as healthy, 461 (23.7%) as unhealthy, and 1242 (63.7%) as regular. Food environments comprised 1441 stores (73.9%), 366 restauration (18.8%), 136 street food outlets (7.0%), and six institutional food outlets (0.3%). The two lowest socioeconomic quintiles (Q1–Q2) exhibited a lower proportion of healthy food outlets (39.4%). Significant positive spatial autocorrelation was observed for stores, restaurants, and street food outlets (*p* < 0.005). Conclusions: Food environments were unequally distributed, with a higher density of healthy food outlets in areas characterized by greater population density and higher socioeconomic status. The observed spatial autocorrelation highlights the need for targeted public health interventions to reduce inequalities in access to healthy foods. These findings provide evidence to support policies aimed at promoting more equitable and healthier food environments in medium-sized cities.

## 1. Introduction

Chile ranks among the Organization for Economic Co-operation and Development (OECD) countries with the highest prevalence of excess weight, encompassing both overweight and obesity [1]. Over 75% of individuals aged 15 years and older are affected [2], and nearly 50% of children entering primary school are classified as overweight or obese [3]. Despite the significance of this public health issue, Chile allocates one of the lowest per capita expenditures on obesity-related healthcare among OECD countries, with USD PPP 102.8 per capita, compared to the OECD average of USD PPP 209.5 [1]. The rising prevalence of obesity, diet-related chronic diseases, and premature mortality represents a common challenge in many countries [4,5]. This issue is strongly influenced by the interaction between food environment factors and social determinants of health [6]. An obesogenic food environment has emerged, which is characterized by the high availability of ultra-processed foods and the limited availability of healthy or natural food options [7,8]. Food environments (FE) are defined as the settings where the physical presence or absence of food and beverages, the proximity of food supply points, geographic distribution, and road networks facilitating access to these points of sale can influence food and beverage choices, thereby affecting individuals’ nutritional status. Additional factors, such as food advertising and pricing, are also associated with the concept of FE and may further shape consumption decisions [9].

FE are recognized as key structural determinants of dietary behaviors, influencing food choices through availability, accessibility, and exposure to different types of foods, and has been identified as a critical factor in the prevention of obesity and other diet-related non-communicable diseases [10]. Evidence suggests that these environments shape dietary habits and contribute to the development of obesity and related health outcomes. Furthermore, disparities in neighborhood FE are considered part of broader structural determinants that influence health inequalities, including obesity prevalence. Therefore, understanding the spatial distribution of FE is essential to better characterize contextual factors that may influence dietary patterns and population health.

An increasing number of studies have investigated the impact of FE on population eating behaviors. Various methodologies have been employed, including analyzing the geographic location of food and beverage outlets and measuring the distance between individuals’ locations (e.g., home, school, university, workplace, or health centers) and points of sale (e.g., supermarkets, markets, convenience stores). These approaches have been used to assess both the distance and the density of food outlets in the territory [10,11]. However, despite growing evidence on FE, most studies have focused on large metropolitan areas, limiting the understanding of how these environments operate in mid-sized cities.

These cities present distinct territorial and population dynamics, including differences in urban continuity, population density, and access to food outlets. In addition, in Latin American contexts, food environments are shaped by specific socioeconomic and urban characteristics, which may not be adequately captured by studies conducted in large metropolitan settings. Addressing this gap is essential to better inform context-specific public health and nutrition policies.

Another approach to measuring FE involves assessing individuals’ perceptions of the availability of healthy and unhealthy foods within their surroundings, as well as cultural influences and food preferences [12,13]. Areas with a lack of availability and/or variety of healthy foods, as well as areas without access to foods, are defined as food deserts [9]; areas with an overabundance of unhealthy foods and a little access to healthy foods are defined as food swamps [14]. In the United States, counties with high food desert scores are associated with a 77% increase in obesity-related cancer mortality (AOR: 1.59; 95% CI: 1.29–1.94). Similarly, areas with high food swamp scores exhibit the same risk (AOR: 1.77; 95% CI: 1.43–2.19) [15]. However, to comprehensively assess the impact of the environment on the population, it is essential not only to determine the available food supply but also to analyze its territorial distribution and the geographical disparities that influence access to healthy food. Understanding these spatial patterns is essential to inform public health and nutrition policies aimed at reducing inequalities in access to healthy food. To our knowledge, this study is among the first to provide a comprehensive, field-based assessment of FE across two territorially continuous mid-sized cities in Chile.

Therefore, this study aims to analyze the spatial distribution and classification of food outlets in two mid-sized cities in southern Chile, based on food availability, variety, and healthy food advertising. Furthermore, it examines the association between the spatial distribution of these outlets and area-level sociodemographic characteristics, including socioeconomic status and population density, as well as spatial clustering patterns across the territory. Based on the study objectives, we hypothesized that the spatial distribution of food outlets would not be random and that food outlet classification would differ according to area-level socioeconomic status and population density.

## 2. Materials and Methods

### 2.1. Study Design and Setting

The present study is part of the project titled “Exposure to Food Environments and Diet Quality in Schoolchildren with Obesity and Normal Weight in the Ñuble Region, Chile”, funded by the National Agency for Research and Development (ANID) (FONIS SA 18I0127). One of the objectives of this project was to evaluate food environments in the cities of Chillán and Chillán Viejo, Chile, using a set of validated instruments [16] to measure the food environments identified by the Chilean Ministry of Health, including stores, restaurants, institutions, and street food vendors [17]. Both cities are considered medium-sized, with populations of fewer than 200,000 inhabitants; this research covered all the urban areas of both cities on-site [18], which are situated in a continuous territory.

This research employed a cross-sectional observational design, which was approved by the Bioethics and Biosafety Committee of the University of Bío-Bío, 18 October 2018.

### 2.2. Study Area, Sample, and Data Collection

The study focused on food environments within a 400 m radius of the homes and schools attended by a randomly selected sample of 253 schoolchildren from nine public schools. Between September and December 2019, a total of 1949 food outlets were evaluated using specific audit tools for each domain of the FE: stores, restaurants, street food vendors, and institutional food environments [16] (Table 1).

Data collection was carried out by professionally trained nutritionists. A five-day training program was conducted via videoconference, which included a thorough review of the instruments. The application process involved walking through the city streets to directly observe food outlets within a 400 m radius of the initially identified geographic points (home and school). The georeferencing was based on coordinates extracted from the Google Earth platform [19], which allowed for the observation of the geospatial distribution of schools using a projected coordinate system (latitude and longitude). The unit of analysis in this study corresponds to individual food outlets.

### 2.3. Food Environment Assessment and Classification

The study measured, for each food outlet, three dimensions within each of the four instruments developed to analyze FE: availability, variety, and food advertising of healthy foods [16]. Four out of five types of FE, as defined in Chile’s National Food and Nutrition Policy, were used: institutional outlets (kiosks and cafeterias within schools), street food or informal markets (mobile food outlets), stores or formal markets (supermarkets, local markets, etc.), and restauration (restaurants, cafeterias, etc.) [20]. For the institutional FE at schools, only kiosks were assessed. More than 80.0% of schoolchildren participate in the School Feeding Program (PAE), which provides a standardized lunch for all students. The PAE offers a healthy meal that does not include foods with Front-of-Package (FOP) labels [11].

The analysis of the evaluated dimensions (availability of healthy and unhealthy foods, variety of healthy foods and food advertising of healthy foods) was based on binary responses: positive (yes) responses were scored as 1, and negative (no) responses were scored as 0. The sum of the scores was categorized according to quartiles ranges, based on the distribution within each food environment. Scores were classified into three categories for each dimension (availability of healthy and unhealthy foods, variety of healthy foods, and food advertising of healthy foods): unhealthy (scores below the 25th percentile), regular (between the 25th and 75th percentiles), and healthy (above the 75th percentile) [16].

### 2.4. Sociodemographic Variables

To evaluate the relationship between the distribution of food outlets and sociodemographic variables, data from the Socio-Material and Territorial Index (SMTI), developed by the Open Data Center/City Observatory UC, were used [21]. This index is constructed based on census zones and data from the 2017 national census and incorporates four measures: educational level of the head of household, housing materiality, overcrowding, and informal housing arrangements. The SMTI provides a continuous score that was categorized into four quantiles (Q1:Q4), where Q1 represents the lowest socioeconomic level and Q4 represents the highest.

Additionally, population density was calculated at the census tract level and also categorized into quartiles.

### 2.5. Statistical and Spatial Analysis

Statistical analyses were performed to assess the association between food outlet classification, sociodemographic variables, and population density. All food outlets (stores, restauration, street food) and institutional ones were included in the analysis, except those with missing coordinates (in a single case), or those located outside the cities of Chillán and Chillán Viejo (n = 22).

Food outlets were aggregated at the census tracts level and analyzed according to their classification, the population density area and its socio-economic level (SMTI). To assess possible differences between classification, Socioeconomic Status (SES), and population density, we used a Fisher’s exact test to compare overall frequencies to a theoretical distribution as a goodness of fit.

Spatial autocorrelation was assessed using Moran’s Global Index (Moran’s I) for each FE and establishment classification. Moran’s I values range from −1 to +1, with values closer to +1 indicating stronger spatial autocorrelation and suggesting a non-random distribution. Statistical significance was determined through hypothesis testing [22]. We also employed the Local Indicators of Spatial Association (LISA) index to identify spatial clusters. LISA relates the values of each census tract to those of neighboring areas, enabling the identification of tracts with high values surrounded by other high values (high–high), tracts with low values surrounded by other low values (low–low), and tracts with mixed values (high–low and low–high). This indicator was calculated for all study areas and all classifications [23].

Statistical analyses, tables and maps were conducted using R software, version 4.3.1 (R Core Team, Vienna, Austria). A significance level of <0.05 was considered for all statistical analyses.

## 3. Results

The on-site assessment food outlets initially resulted in 1972 locations registered in the original database (non-spatial). After cleaning the database 1949 locations were retained for analysis (spatial data). These were mapped and food environment was identified as follows: 1441 stores (73.93%), six institutional outlets (0.3%), 366 restauration (18.77%), and 138 street food outlets (7.08%) in the cities of Chillán and Chillán Viejo (Table 1). Most stores and street food were classified as regular (69.56% and 60.14%), considering the availability of healthy and unhealthy foods, variety, and advertising of healthy foods; most of the institutional outlets and restauration were classified as unhealthy (50.0% and 54.37%).

**Table 1:** Description of the classification and mapping integration of the different evaluated food environments.

Food Environment	Classification of Outlets	Non-Spatial	Spatial
		n (%)	n (%)
Store	Healthy	205 (14.02)	193 (13.39)
	Regular	1017 (69.56)	1008 (69.95)
	Unhealthy	240 (16.41)	240 (16.65)
	Total	1462 (100.0)	1441 (100.0)
Institutional	Healthy	1 (16.6)	1 (16.6)
	Regular	2 (33.3)	2 (33.3)
	Unhealthy	3 (50.0)	3 (50.0)
	Total	6 (100.0)	6 (100.0)
Restauration	Healthy	17 (4.64)	17 (4.64)
	Regular	150 (40.98)	150 (40.98)
	Unhealthy	199 (54.37)	199 (54.37)
	Total	366 (100.0)	366 (100.0)
Street Food	Healthy	35 (25.36)	35 (25.73)
	Regular	83 (60.14)	82 (60.29)
	Unhealthy	20 (14.49)	19 (13.97)
	Total	138 (100.0)	136 (100.0)
Total	-	1972 (100.00)	1949 (100.00)

According to population distribution, in areas with low population density (<1308 inhabitants), unhealthy food outlets predominated, and only stores were observed (n = 7); most of these were classified as unhealthy (n = 4; 57.1%). In medium–low population areas (1308–2138 inhabitants), most stores were classified as regular (n = 415; 74.7%). Among restauration, 40.08% were classified as unhealthy (n = 93), while 32.8% of street food outlets were classified as healthy (n = 21). In areas with medium–high population density, most stores were classified as regular (n = 247; 86.97%). Among restauration, 44.44% (n = 48) were classified as unhealthy; 6.48% (n = 7) of street food outlets were classified as unhealthy. Finally, in areas with higher population density (>3478 inhabitants), 88.37% (n = 114) of stores were classified as healthy and 49.57% of restauration (n = 58) was classified as unhealthy (Table 2).

The medium–high socioeconomic level has the highest proportion of food outlets classified as unhealthy (n = 182; 39.47%). A noteworthy aspect is that 43.40% (n = 79) of the restauration food environment on this socioeconomical level was unhealthy. As socioeconomic status increases from low to high, the number of food retail outlets classified as healthy also increases (Q1–Q2 n = 97; Q3–Q4 n= 149) (Table 3).

**Figure 1:**
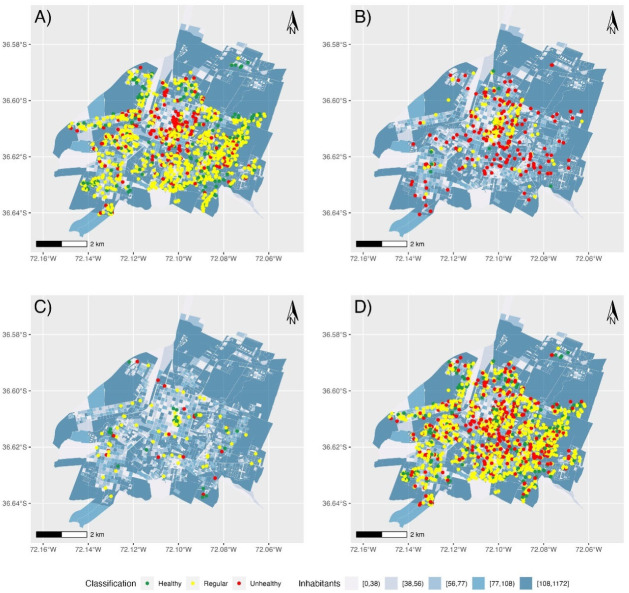
Distribution of the classification of the food outlets by each food environment and population density: (**A**) store; (**B**) restauration; (**C**) street food; and (**D**) all food outlets.

By analyzing the distribution of food outlets in the area according to food environment, classification of food outlets, and population density, patterns of distribution emerge with a high density of food outlets classified in the worst category (Figure 1; unhealthy, red color), independently of the food environment analyzed.

According to Figure 1, it can be observed that the central area of this territory shows a high availability of food outlets, predominantly offering unhealthy food options (red circle), with limited availability and variety of healthy foods (green circle). Furthermore, there is an absence of advertising for healthy food options that were considered in the final score [16].

To evaluate the spatial autocorrelation, the Global Moran Index (Moran’s I) was used; significant differences were evaluated with a chi-square test (Monte Carlo simulation). As Moran’s I value increases (>0), it indicates positive autocorrelation and spatial clustering. We observed a positive and statistically significant spatial autocorrelation in stores (Moran’s I: 0.16; *p*-value: <0.001), Restauration (Moran’s I: 0.38; *p*-value: <0.001), and Street Food (Moran’s I: 0.08; *p*-value: <0.01) (Table S1).

**Figure 2:**
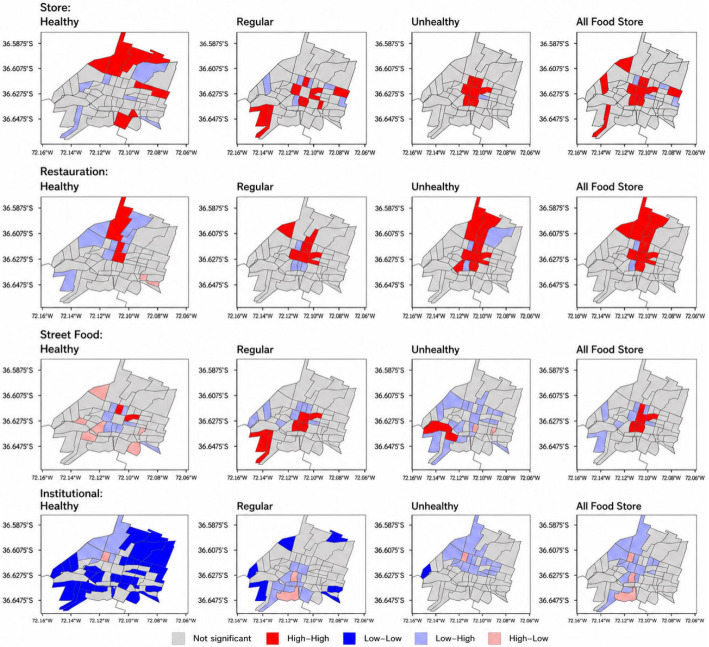
Spatial distribution of food outlets by classification within each food environment highlighting high and low concentration zones and obesogenic areas in the analyzed cities.

Figure 2 illustrates the LISA results showing that red areas represent zones with a high number of food outlets, surrounded by other areas with a similarly high number (high–high) of food outlets. In contrast, blue areas (low–low) indicate zones with few food outlets, surrounded by areas with a similarly low number of food outlets. The remaining categories represent intermediate values. It can be observed that food outlets in the stores and restauration food environments classified as unhealthy are concentrated in the city’s central area, further characterizing these areas with a high concentration of unhealthy food outlets. The institutional dimension represents spatial heterogeneity due to the low number of food outlets.

## 4. Discussion

Our results demonstrate the presence of areas with a high presence of unhealthy food outlets with a low availability and limited variety of healthy foods in the cities of Chillán and Chillán Viejo, medium-sized cities located in southern Chile. These two cities form an area that overlaps in a single territory, where it is evident that the northern area lacks food outlet options. The findings support the proposed hypothesis, indicating that the spatial distribution of food outlets varies according to area-level socioeconomic status and population density. Areas characterized by higher socioeconomic status and greater population density tend to have a higher proportion of food outlets classified as healthy.

Therefore, we identified specific areas within the territory that exhibited characteristics of food deserts, evidenced by low availability of establishments offering healthy foods, limited variety in the food options available, and an absence of promotional strategies for nutritionally adequate options.

These findings suggest inequalities in access to healthy foods within the study area, which could have implications for the configuration of the food environment and the consumption patterns of the population. To the best of our knowledge, this study is the first-ever mapping of all food environments in a large territorial area (two medium-sized cities) in the country, using primary data collection and classifying food outlet establishments.

Identifying food deserts is a complex task that can be approached using different methodological strategies. Many studies typically rely on secondary data to identify and classify food outlets; however, this approach has the limitation of not capturing important dimensions of the food environment such as food streets or the presence of food advertising at food outlets. Recent studies reinforce that secondary data must be used with caution, principally “for the characterization of areas with low socio-economic status”, principally in rural areas [24,25].

The main reasons are related to the inconsistency of information obtained from the official records of food outlets. These databases often have a lag of up to two years; often, food outlets are no longer present in the territory. In addition, official databases tend to focus exclusively on formal establishments, excluding informal commerce and small-scale sales, which constitute a significant part of the food supply in many communities. This omission is particularly relevant in Latin America, where informal markets play a key role in food availability and accessibility [26,27,28]. In our study, we conducted direct primary data collection in the field, enabling us to overcome some of the limitations associated with the use of secondary data.

The presence of food deserts is associated with the risk of food insecurity [29] and chronic diseases. In the United States, living in a food desert was associated with cardiovascular health risk (range 0–14) (β = 0.048; *p* < 0.01), with a high influence by residence area and personal socioeconomic status [30]. In a recent systematic review, Key et al. (2023) could not find an association between an increase in BMI in children and the fact of living in a food desert [31].

Obesity is a complex disease, influenced by many factors; the presence of food deserts surrounding homes does not necessarily determine this condition. The analysis of 18,381 households located in 2104 US counties in the United States demonstrated that some food environment characteristics, like the presence of food deserts, were associated with obesity when controlled by food environment factors at the individual, household, and neighborhood levels [32].

Population density influences the presence of food outlets [33]. In the analyzed territory in this study, areas with fewer than 1308 inhabitants had no food outlets classified as healthy. In contrast, areas with higher population density exhibited a greater concentration of food outlets (stores, restauration, and street foods). This highlights the inequity related to the socioeconomic level of the population living in the territory, where areas with higher poverty levels have a lower proportion of healthy food outlets, while areas with higher population density have a greater density of food outlets.

Similar findings were reported in Seattle, United States, where socioeconomic level and ethnicity influenced the availability of healthy foods. Areas with lower income and a larger proportion of black residents had lower healthy food availability scores compared to areas with higher incomes (8.06 [95% CI. 7.04–9.07] vs. 12.40 [95% CI, 10.63–14.17], *p* < 0.001) [34].

The lack of availability of food outlets by population density and socioeconomic level in the territories, such as those observed in Chillán and Chillán Viejo, highlights the urgent need to rethink policies aimed at improving access to a healthy food environment in Chile. In recent decades, Chile has implemented a series of policies to enhance the nutritional quality of foods [35]; however, few of these policies address the need to improve the physical and economic availability of healthy foods, considering the geographical distribution. The central area of this territory presents mostly food outlets classified as unhealthy; the peripheric areas present a lack of food outlets, indicating social and geographical disparities in the food outlets.

One limitation of this study is that it did not consider the mobility of individuals to other places where they might acquire food, which could significantly influence their actual access to healthy or unhealthy food environments. Additionally, the analysis of the digital food environment was not incorporated, despite its growing importance in recent years due to the rise of online shopping platforms and food delivery services. Failing to consider these aspects may underestimate or overestimate the influence of the evaluated food environments. Future research should further explore aspects related to population mobility for food purchasing, such as the distance that individuals must travel from their homes to food retail outlets, particularly those considered as healthier. Furthermore, greater attention should be given to understanding the influence of the digital food environment on food acquisition behaviors.

These results present an opportunity to intervene in the territory by promoting new types of healthy food outlets with affordable prices, such as local food markets, and restructuring existing food outlets to increase the availability of healthy foods. This is particularly relevant considering that the Chillán and Chillán Viejo region is a major producer of fruits and vegetables in Chile. Another aspect worth highlighting is the high cost of healthy eating in the Americas region, where Chile is no exception. According to the FAO in its report Cost and Affordability of a Healthy Diet (CoAHD), the cost of a healthy diet in Chile is USD PPP 4.54 (dollar per person per day), while in Brazil, it is USD PPP 4.45, and in the United States, it is USD PPP 2.63 [36].

Another limitation observed was the underrepresentation of street food outlets, influenced by the data collection period, which occurred at the beginning of the COVID-19 pandemic. These results do not represent a national context but provide valuable evidence of food environments in Chile.

## 5. Conclusions

This study revealed a heterogeneous spatial distribution of food outlets across two medium-sized cities in southern Chile, with clustering patterns that varied according to outlet type, population density, and area-level socioeconomic status. The findings indicate that the spatial distribution of food outlets is associated with both population density and socioeconomic status, with a higher proportion of outlets classified as healthy located in areas characterized by higher socioeconomic status and greater population density. In addition, food outlets overall tended to be concentrated in central urban areas.

These results suggest that the local food environment is shaped by a combination of territorial and contextual factors rather than by a uniform socioeconomic gradient. Consequently, policies and interventions aimed at improving access to healthy food options should take into account spatial disparities in the food environment, distinguishing between the availability of healthy food outlets, the concentration of less healthy outlets, and patterns of urban clustering. Such evidence may contribute to the development of more targeted and equitable strategies for promoting healthier urban food environments.

## Figures and Tables

**Table 2:** Distribution of food outlets classification in each food environment by population density.

		Classification of Food Outlets			
Population Density	Food Environment Dimension	Healthy n (%)	Regular n (%)	Unhealthy n (%)	Total n (%)
Low (<1308 inhabitants)	Store	0	3 (1)	4 (57.1)	7 (100.0)
	Institutional	0	0	0	0
	Restauration	0	0	0	0
	Stret Food	0	0	0	0
	Total	0	3 (42.9)	4 (57.1)	7 (100.0)
Medium–Low (1308–2138 inhabitants)	Store	34 (53.1)	415 (74.7)	133 (57.32)	582 (68.39)
	Institutional	1 (1.56)	1 (0.18)	2 (0.86)	4 (0.47)
	Restauration	8 (12.5)	99 (17.83)	93 (40.08)	200 (23.50)
	Stret Food	21 (32.8)	40 (7.2)	4 (1.72)	65 (7.6)
	Total	64 (100.0)	555 (100.0)	232 (100.0)	851 (100.0) *
Medium–High (2139–3478 inhabitants)	Store	45 (84.90)	247 (86.97)	53 (49.07)	345 (77.52)
	Institutional	0	0	0	0
	Restauration	5 (9.43)	19 (6.69)	48 (44.44)	72 (16.17)
	Stret Food	3 (5.66)	18 (6.33)	7 (6.48)	28 (6.29)
	Total	53 (100.0)	284 (100.0)	108 (100.0)	445 (100.0) *
High (>3478 inhabitants)	Store	114 (88.37)	343 (85.75)	50 (42.73)	507 (78.48)
	Institutional	0	1 (0.25)	1 (0.85)	2 (0.30)
	Restauration	4 (3.10)	32 (8.0)	58 (49.57)	94 (14.55)
	Stret Food	11 (8.52)	24 (6.0)	8 (6.83)	43 (6.65)
	Total	129 (100.0)	400 (100.0)	117 (100.0)	646 (100.0) *
Total		246 (100.0)	1242 (100.0)	461 (100.0)	1949 (100.0)

* Fisher test with a *p*-value < 0.05.

**Table 3:** Distribution of food outlet classification in each dimension by socioeconomic level of the area.

		Classification of Food Outlets			
Socioeconomic Level	Food Environment Dimension	Healthy n (%)	Regular n (%)	Unhealthy n (%)	Total n (%)
Low (Q1)	Store	5 (100.0)	35 (94.59)	5 (62.5)	45 (90.0)
	Institutional	0	0	3 (37.5)	3 (6.0)
	Restauration	0	2 (5.40)	0	2 (4.0)
	Stret Food	0	0	0	0
	Total	5 (100,0)	37 (100.0)	8 (100.0)	50 (100.0) *
Medium–Low (Q2)	Store	32 (86.48)	140 (92.71)	18 (62.06)	190 (87.55)
	Institutional	0	3 (1.98)	8 (27.58)	11 (5.06)
	Restauration	5 (13.51)	8 (5.29)	3 (10.34)	16 (7.37)
	Stret Food	0	0	0	0
	Total	37 (100.0)	151 (100.0)	29 (100.0)	217 (100.0) *
Medium–High (Q3)	Store	54 (85.71)	288 (90.28)	53 (47.32)	395 (79.95)
	Institutional	3 (4.76)	12 (3.76)	54 (48.21)	69 (13.96)
	Restauration	6 (9.52)	19 (5.95)	5 (4.46)	30 (6.0)
	Stret Food	0	0	0	0
	Total	63 (100.0)	319 (100.0)	112 (100.0)	494 (100.0) *
High (Q4)	Store	102 (72.34)	545 (74.14)	164 (52.56)	811 (68.26)
	Institutional	14 (9.92)	135 (18.36)	134 (42.94)	283 (23.82)
	Restauration	24 (17.02)	53 (7.21)	11 (3.52)	88 (7.40)
	Stret Food	1 (0.70)	2 (0.27)	3 (0.96)	6 (0.50)
	Total	141 (100.0)	735 (100.0)	312 (100.0)	1188 (100.0) *
Total		246 (100,0)	1242 (100.0)	461 (100.0)	1949 (100.0)

* Fisher test with a *p*-value < 0.05. Q1: quartile 1; Q2: quartile 2; Q3: quartile 3; Q4: quartile 4.

## Data Availability

The datasets are not publicly available because they contain sensitive information, including the geolocation of commercial food establishments. Public dissemination of these data was not authorized by the Research Ethics Committee under the approved study protocol.

## Supplementary Materials

The following supporting information can be downloaded at: https://www.mdpi.com/article/10.3390/nu18132131/s1, Table S1: Results of Moran’s Index by type of food environment and classification.

## Abbreviations

The following abbreviations are used in this manuscript:

FE	Food environments
PAE	School Feeding Program
FOP	Front-of-Package
OECD	Organization for Economic Co-operation and Development
ANID	Agency for Research and Development
OCUC	Observatorio de Ciudades UC
SMTI	Socio-Material and Territorial Index
LISA	Local Indicators of Spatial Association
CoAHD	Cost and Affordability of a Healthy Diet
