# Supplementary material for: Geospatial Distribution of Food Environments and Their Association with Sociodemographic Factors in Two Mid-Sized Chilean Cities
Source: Nutrients. 2026 Jul 1;18(13):2131. doi: 10.3390/nu18132131 (PMC13363557; doi:10.3390/nu18132131)
Supplement: Supplementary file 1 [file nutrients-18-02131-s001.zip › nutrients-4239405-supplementary.pdf]

Supplementary Materials

Table S1. Results of the Moran's Index by type of food environment and its classification

Food environment	Classification	Moran's I	p-value**
Store	Healthy	0.3134	0.0000
	Regular	0.0982	0.0004
	Unhealthy	0.3300	0.0000
	Total	0.1569	0.0000
Institutional	Healthy	-0.0106*	
	Regular	-0.0170	0.5655
	Unhealthy	0.0755	0.0916
	Total	-0.0041	0.4743
Restauration	Healthy	0.1617	0.0057
	Regular	0.3038	0.0000
	Unhealthy	0.4159	0.0000
	Total	0.3769	0.0000
Street Food	Healthy	0.0097	0.2171
	Regular	0.0675	0.0047
	Unhealthy	0.0730	0.1371
	Total	0.0758	0.0021

* Only one Institutional Food Environment was considered Healthy; ** Chi- square (Monte Carlo simulation)
